# Long-term patency rate of the translocated autologous saphenous vein versus prosthetic material in vascular access surgery for haemodialysis and parenteral nutrition

**DOI:** 10.1177/11297298211013133

**Published:** 2021-11-30

**Authors:** Wouter Driessen, Wilbert van der Meijden, Geert Wanten, Frank van Hoek

**Affiliations:** 1 Department of Vascular surgery, Radboud University Medical Center, Nijmegen, Gelderland, The Netherlands; 2 Department of Nephrology, Radboud University Medical Center, Nijmegen, Gelderland, The Netherlands; 3 Department of Gastroenterology and Hepatology, Radboud University Medical Center, Nijmegen, Gelderland, The Netherlands

**Keywords:** Vascular access, arteriovenous fistula, arteriovenous angioaccess, saphenous vein, PTFE, graft patency, access related complications, haemodialysis, home parenteral nutrition, survival analysis

## Abstract

**Objective:**

To evaluate the long-term patency rate of the arteriovenous angioaccess (AVA) with interposition of either autologous or prosthetic material as a last option for vascular access in the upper extremity.

**Methods:**

This is a retrospective chart review study of all patients who received an AVA with autologous saphenous vein (SV Group, *n* = 38) or prosthetic material (PTFE Group, *n* = 25) as a conduit from the year 1996 to 2020 in the Radboud University Medical Center (Radboudumc). Data were retrospectively extracted from two prospectively updated local databases for vascular access, one for haemodialysis (HD) and one for parenteral nutrition (PN). When required, the medical records of each patient were used. Data were eventually collected anonymously and analysed in SPSS 25. Kaplan-Meier life-tables were used for the statistical analysis.

**Results:**

Primary patency at 12 and 48 months was 30% and 20% in the SV group and 45% and 14% in the PTFE group. No significant difference was shown in the median primary patency rate (*p* = 0.715). Secondary patency at 12 and 48 months was 63% and 39% in the SV group and 55% and 19% in the PTFE group. This was considered a significant difference in median secondary patency in favour of the SV with 41.16 ± 17.67 months against 13.77 ± 10.22 months for PTFE (*p* = 0.032). The incidence of infection was significantly lower in the SV group (*p* = 0.0002). A Kaplan-Meier curve could not detect a significant difference in secondary patency between the access for haemodialysis and the access for parenteral nutrition. The secondary patency of the SV in parenteral nutrition access, was significantly higher when compared with PTFE (*p* = 0.004).

**Conclusion:**

The SV can be preferred over PTFE when conduit material is needed for long-term vascular access for HD or PN treatment due to its higher secondary patency and lower infection risk.

## Introduction

A vascular access is a lifeline for patients with end-stage kidney disease (ESKD) who need haemodialysis (HD)^1^ and in rare cases of chronic intestinal failure (CIF) who need parenteral nutrition (PN).^2^ For short-term treatment central venous catheters (CVCs) are often used as vascular access, but because of high infection rates of the CVCs in both ESKD patients^3–6^ and CIF patients^7–10^ the arteriovenous fistula (AVF) is usually preferred when treatment is expected to be long-term or life-long.^11^

The AVF is a surgical connection between the arterial and venous system. The goal is to create an accessible vascular structure with sufficient blood flow that can be cannulated repeatedly to permit adequate HD or PN treatment.

As is published in the European Best Practice guidelines,^11^ the primary choice for an AVF is the autogenous radio-cephalic AVF (RCAVF). When the quality of these peripheral vessels are insufficient, more proximal fistulae as the brachio-cephalic AVF (BCAVF) or the brachio-basilic AVF (BBAVF) are indicated at the elbow and upper-arm region.

When these options are impossible or the access has failed, graft implants as a vascular conduit can be considered to construct an AVA. This technique uses looped or straight prosthetic materials, mostly PTFE, that function as a conduit between artery and vein.

An alternative to the prosthetic material is the use of an autologous transposed vein. Most commonly used for this procedure is the great saphenous vein (GSV).

### Rationale

The Radboudumc uses the GSV since 1997 when the RCAVF, BCAVF and BBAVF are impossible or have failed. As one of few in the world the Radboudumc uses this technique not only for the indication of HD but also when long-term PN treatment is needed. The primary objective of this retrospective chart review will be to analyse these cases and compare them with the prosthetic conduits. The secondary objective of this study is to compare the HD AVA with the PN AVA. The results will provide an overview of the quality of the AVA with autologous vein interposition in both patient groups. Ultimately this can lead to the identification of significant factors affecting the patency, infection risk and maybe lead to changes in the protocol of vascular access surgery.

## Methods

### Study design

This retrospective chart review study identified all patients who received an AVA with an autologous vein or prosthetic material as a conduit from the year 1996 to 2020. Data were retrospectively extracted from two prospectively updated local databases for vascular access. One database contained all accesses intended for haemodialysis and one for parenteral nutrition. When required, the medical records were used to complement the final data.

This study has been performed according to the local research ethics committee guidelines in such way no informed consent was necessary.

### Study population

A total of 46 patients were included with 63 AVAs. There were 25 patients with 31 SV conduits, 14 patients with 17 PTFE conduits and seven patients with both types of conduit material (eight PTFE and seven SV). Of the 38 SV conduits, 37 were GSV and one was small saphenous vein (SSV). Of the 25 PTFE conduits, 24 were GORE-TEX^®^ and one Rapidax™. All conduits were interposed between artery and vein in the upper extremity. According to a Chi-square test, there was a significantly uneven distribution of AVAs of patients with diabetes mellitus and overweight and access indication (Table 1).

**Table 1.** Preoperative demographics and comorbidity of the patient groups.

	Autologous conduit (*n* = 38, 60.3%)	Prosthetic conduit (*n* = 25, 39.7%)	*p*
Age, years	52 ± 12	51 ± 11	0.624
Female, *n* (%)	24 (63.2)	16 (64.0)	0.946
Hypertension, *n* (%)	17 (44.7)	17 (68.0)	0.070
Diabetic mellitus, *n* (%)	2 (5.3)	9 (36.0)	0.002
Tobacco use, *n* (%)	7 (18.4)	7 (28.0)	0.371
PAOD, *n* (%)	8 (21.1)	9 (36.0)	0.191
Overweight, *n* (%)	8 (21.1)	12 (48.0)	0.025
HD indication, *n* (%)	14 (36.8)	16 (64.0)	0.035

Age is presented as mean ± SD, the rest is presented as number of patients and percentages in brackets.

PAOD: peripheral arterial occlusion disease; Overweight – defined as BMI ⩾ 25; HD indication – percentage of haemodialysis access.

There were 22 patients with 30 HD AVAs and 24 patients with 33 PN AVAs. HD AVAs were accessed three to four times a week for haemodialysis therapy and received heparin during the session. PN AVAs were accessed three to seven times a week for nutrition fluid infusion and the patients were treated with intravenous anticoagulant medication warfarin. Intravenous administration is used, because of malabsorption due to short bowel syndrome.

### Study protocol

Data were collected from two local databases, one for dialysis access and one for PN access. These data were complemented by a targeted search in the electronical medical records to determine, baseline patient characteristics; shunt characteristics, including primary-, primary assisted- and secondary patency, defined according to consensus reporting standards;^12^ and rates of post-operative complications. Perioperative- and postoperative failures within 3 months were included in all analysis.

All patients in the Radboudumc who received vascular access surgery were preoperative evaluated through colour duplex ultrasound (CDU) to determine the best option for anastomoses. Once the RCAVF, BCAVF and BBAVF were not an option, the GSV was assessed through CDU. This vessel was considered sufficient when the diameter was >3 mm when proximal manually compressed. When this was met, the vessel was used as conduit material. If the GSV was not sufficient, prosthetic materials were used.

Six weeks postoperative, patients were being evaluated through physical and CDU examination by the vascular surgeon. Also, the HD patients who were treated at the centre receive Transonic bloodflow measurements every 3 months during HD treatment. When the HD treatment was at home, the patient was evaluated every 6 months through CDU. When indicated, both HD and PN patients received CDU examination.

### Surgical technique

CDU examination was used to identify the most sufficient artery and vein for the anastomotic site in the upper extremity and the length between both sites was measured. Next, the saphenous vein was harvested via an open technique. Starting from the saphenofemoral junction, the saphenous vein was prepared to distal and sections of the vein were performed to correspond the length between the anastomotic sites in the upper extremity.

### Statistical analysis

Data were collected anonymously and analysed in SPSS 25. For analysing the homogeneity of both patient groups Independent Samples *T*-tests were used for continuous variables and Chi-square-tests for categorical variables. The same analyses were used for the occurrence of complications related to the AVA.

The outcome of this study was the survival of the AVA, expressed in primary-, assisted primary- and secondary patency rate. Kaplan-Meier curves were used to describe these patency rates. Log-rank tests were used to estimate differences between the conduit materials. Cox Regression analysis was used to compensate for confounders.

*p*-Values less than 0.05 were considered statistically significant.

## Results

A total of 63 AVAs were included for all analyses, 38 with SV and 25 with PTFE. In 33 cases, occlusion was the reason for failure. Eight patients died with a functional AVA. Seven AVAs were abandoned because of dysfunction and puncture problems, which was due to fibroses of the GSV in four cases, persistent PN pump alarms without reason in two cases and high intraluminal pressure making nutrition fluid infusion impossible in one case. Four AVAs were still functional at the end of this study. Another four were abandoned due to infection. Three AVAs were abandoned due to insufficient flow without possibility for intervention and three patients abandoned their AVA because of kidney transplantation and one was no longer observed after discontinuation of treatment.

The arterial anastomosis was made with the brachial artery in 25 cases of the SV group and 21 cases of the PTFE group. A few times the radial-, axillary- and subclavian artery were used. For the venous anastomosis, the cephalic-, basilic- and brachial vein were mostly used and in a few cases the median cubital-, ulnar-, axillary- and subclavian vein. In four cases both arterial and venous anastomoses were unknown.

For the survival analyses, 11 (28.9%) AVAs were censored in the SV group and 5 (20.0%) in the PTFE group. Reasons for censoring were death of patient, a still functional AVA at the end of the study, kidney transplantation and discontinuation of treatment.

For an overview of the occurrence of AVA related complications, the incidence rate of each complication per 1000 days was used. The incidence rate of infections was significantly higher in the PTFE group (*p* = 0.0002) and the incidence rate of pseudoaneurysms was significantly higher in the SV group (*p* = 0.026). The incidence rate of puncture problems tended to be higher in the PTFE group (*p* = 0.058) and the incidence rate of bleedings tended to be higher in the SV group (*p* = 0.089). AVA failure within 3 weeks, including perioperative- and postoperative failure, occurred in 23.7% of the SV group and 36.0% of the PTFE group, without a significant difference (*p* = 0.290) (Table 2).

**Table 2.** The complications related to the arteriovenous angioaccess.

	Autologous conduit (*n* = 38, 60.3%)	Prosthetic conduit (*n* = 25, 39.7%)	*p*
Thrombosis, IR^a^	9.44 ± 47.13	9.64 ± 39.84	0.891
Stenoses, IR^a^	2.07 ± 2.86	1.20 ± 2.85	0.360
Infection, IR^a^	0.08 ± 0.25	13.42 ± 42.55	0.000
HAIDI, IR^a^	0.13 ± 0.58	0.05 ± 0.25	0.207
Pseudoaneurysm, IR^a^	0.03 ± 0.14	0.00 ± 0.00	0.026
Puncturing problems, IR^a^	0.12 ± 0.43	0.46 ± 2.30	0.058
Bleeding, IR^a^	1.72 ± 8.13	0.32 ± 1.59	0.089
Post-operative complications surgical site SV, *n* (%)^b^	3 (7.9)	0 (0.0)	0.150
Insufficient flow, *n* (%)^b^	9 (23.7)	6 (24.0)	0.977
AVA failure within 3 months, *n* (%)^b^	9 (23.7)	9 (36.0)	0.290

Data are presented as ^a^number of complications per 1000 days ± SD.

b Number of cases and percentages in brackets.

IR: incidence rate; Thrombosis: includes all events of thrombotic occlusions that had successful interventions; Stenoses: includes all events of stenoses that had successful interventions; Infection: surgical site infection; tissue infection; sepsis, HAIDI: haemodialysis access induced distal ischaemia; Puncturing problems: inability to puncture and reason for intervention; Post-operative complications surgical site SV: includes infection, bleeding and haematoma of the surgical site of autologous vein extirpation; Insufficient flow: flow volume of <200 ml/min at the first CDU follow-up; AVA failure within 3 months: includes al AVAs who failed perioperative to 3 months postoperative.

### Primary patency

**Figure 1.**
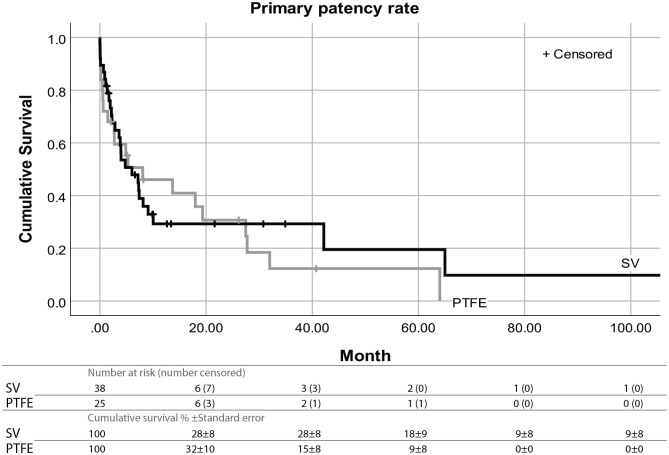
Kaplan-Meier survival curves with Log-rank analysis showing the primary patency over months by conduit material. Log-rank Chi-square value: 0.134; *df*: 1; *p*: 0.715.

Primary patency refers to ‘intervention-free access survival’. The cumulative proportional survival for the SV at 12, 24, 48 and 72 months was 30%, 30%, 20% and 10%. For PTFE it was 45%, 30%, 14% and 0%. With the Kaplan-Meier method, there was no significant difference found in median survival time with 6.03 ± 2.63 months for the SV and 8.06 ± 6.33 months for PTFE (*p* = 0.715) (Figure 1).

### Assisted primary patency

Assisted primary patency refers to ‘thrombosis-free access survival’. The cumulative proportional survival for the SV at 12, 24, 48 and 72 months was 60%, 54%, 38% and 20%. For PTFE it was 53%, 29%, 13% and 0%. According to the Kaplan-Meier method, a Log-rank test found a significant difference in median survival time in favour of the SV with 41.16 ± 17.30 months against 12.83 ± 7.47 months for PTFE (*p* = 0.012).

### Secondary patency

**Figure 2.**
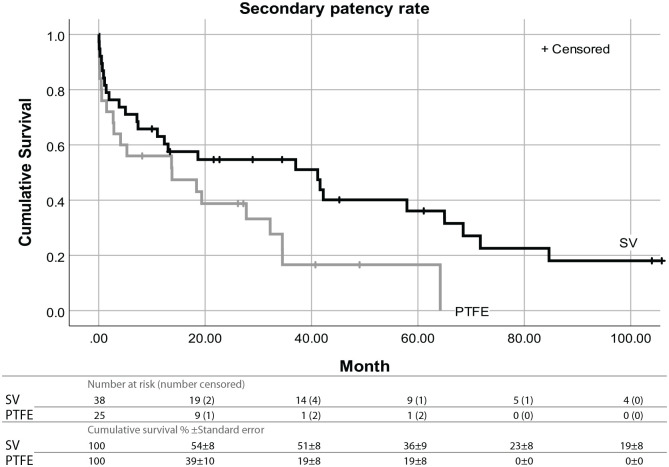
Kaplan-Meier survival curves with Log-rank analysis showing the secondary patency over months by conduit material Log-rank Chi-square value: 4.59; *df*: 1; *p*: 0.032.

Secondary patency refers to ‘access survival until abandonment’. The cumulative proportional survival for the SV at 12, 24, 48 and 72 months was 63%, 54%, 39% and 23%. For PTFE it was 55%, 38%, 19% and 0%. According to the Kaplan-Meier method, a Log-rank test found a significant difference in median survival time in favour of the SV with 41.16 ± 17.67 months against 13.77 ± 10.22 months for PTFE (*p* = 0.032) (Figure 2).

Because of an uneven distribution of AVAs of patients with diabetes mellitus, overweight and AVA indication, a Cox Regression model was executed for these confounders. This model identified AVA indication as a significant covariate affecting the secondary patency (*p* = 0.010). HD indication was considered a positive factor for the AVA patency, with an HR of 0.370. When corrected for AVA indication, the type of conduit remained a significant factor affecting the patency, with the SV positively affecting the secondary patency (Table 3).

**Table 3.** Cox regression model on the secondary patency with Hazard ratio and 95% CI.

	Beta	SE	*p*-Value	HR	95% CI for HR	
					Lower	Upper
Diabetes mellitus	−0.116	0.471	0.806	0.891	0.354	2.241
Overweight	0.425	0.364	0.242	1.530	0.750	3.120
HD indication	−0.994	0.385	0.010	0.370	0.174	0.787
SV conduit	−1.024	0.379	0.007	0.359	0.171	0.755

SE: standard error; HR: hazard ratio; CI: confidence interval; Diabetes mellitus, Overweight, HD indication and SV conduit as covariates.

### Secondary objective

The secondary objective was to compare the PN AVAs (PN group, *n* = 24) with the HD AVAs (HD group, *n* = 14). For this analysis, only the autologous AVA were used for homogeneity.

Of all the AVA related complications, stenoses (*p* = 0.010) occurred more frequently in the HD group and infections (*p* = 0.001) occurred more frequently in the PN group, with zero cases in the HD group. Occlusions (*p* = 0.072), insufficient flow (<200 ml/min) (*p* = 0.067) and AVA failure within 3 months (*p* = 0.067) tended to occur more frequently in the PN group, although not significantly.

**Figure 3.**
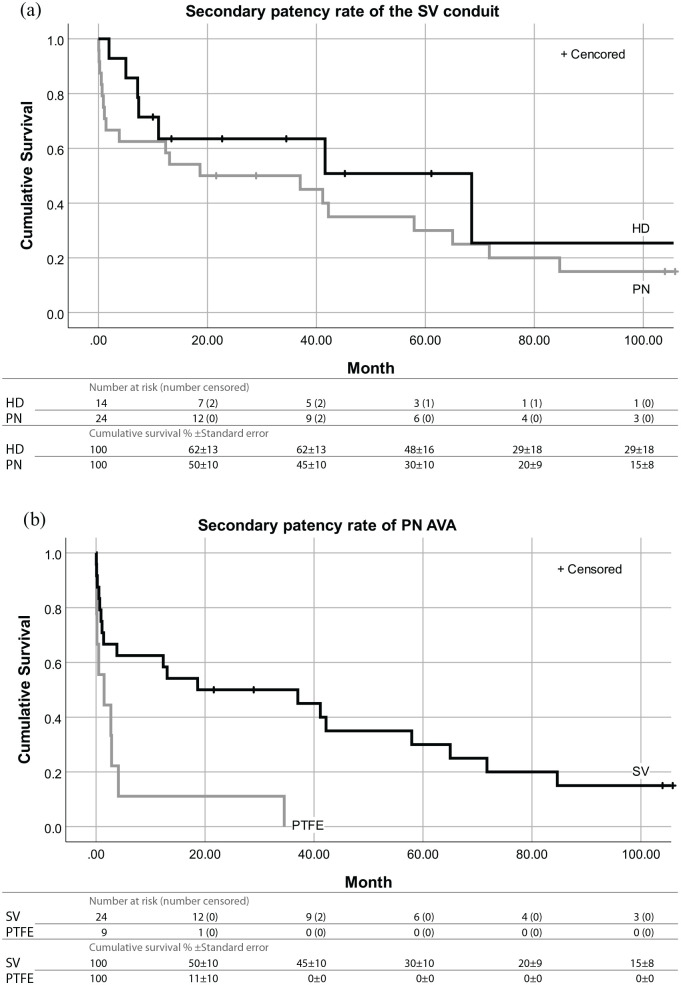
(a) Kaplan-Meier survival curves with Log-rank analysis showing the secondary patency over months of the SV conduit by access indication. Log-rank Chi-square value: 1.108; *df*: 1; *p*: 0.293 and (b) Kaplan-Meier survival curves with Log-rank analysis showing the secondary patency over months of the PN AVA by conduit material. Log-rank Chi-square value: 8.447; *df*: 1; *p*: 0.004.

Time expressed in secondary patency, the cumulative proportion survival of the SV in the HD group at 12, 24, 48 and 72 months was 63%, 63%, 49% and 29%. In the PN group, it was 63%, 50%, 35% and 20%. Although the HD group tended to have a more favourable patency rate, it was not considered significant (*p* = 0.293) (Figure 3(a)).

In another analysis, the conduit material of the PN AVAs were compared (SV conduit, *n* = 24; PTFE conduit, *n* = 9). Stenoses (*p* = 0.017), infections (*p* = 0.000025) and puncture problems (*p* = 0.003) occurred more frequently in PTFE. AVA failure within 3 months occurred in 33.3% of the SV and 77.8% of PTFE, which was considered a significant difference (*p* = 0.022).

When the time was expressed in secondary patency, the cumulative proportion survival of the PN AVAs at 12, 24, 48 and 72 months for the SV was 63%, 50%, 35% and 20%. For PTFE, it was 11%, 11% and at 48 months 0%. This was considered a significant difference with a *p*-value of 0.004 (Figure 3(b)).

## Discussion

To determine the better choice for conduit material, the patency rate was used as the main outcome. The benefit of the SV conduit on the assisted primary patency and secondary patency rate was higher when compared with the PTFE conduit. For the primary patency rate there was no significant benefit for either of the materials.

Literature has shown that the autologous conduit can be preferred due to satisfactory patency rates, low infection risks and cost when compared with synthetic ones.^13–17^ Smith et al.^15^ analysed the patency for the GSV translocation and found similar results to this study in primary patency and secondary patency at 12 and 24 months. Uzun et al.^17^ found higher primary and secondary patency at 12 and 24 months for both SV and PTFE conduits, and a significant difference in favour of the SV. The autologous conduit was made with the below the knee part of the GSV. None of these studies included angioaccesses for PN. Higher primary patency, intervention-free access survival, for comparable conduit materials may be caused by a different approach in follow up to maintain patency. The follow-up period of this study is longer and gives a clear view of the course of the patency rates. Especially long-term, from 24 months, advantage in patency is visible.

In the PTFE group, the number of infections was significantly higher when compared with the SV group. Such difference is not mentioned in previous research of AVA in the upper extremity. But, it is known that synthetic implants compromise host defences and provide a foothold for bacteria.^18^ Other complications occurred with the same frequency. The infection and haematoma at the surgical site of the SV extirpation occurred only in patients of the SV group.

For interpreting the results correctly it is important to take into account that the baseline characteristics were not similar in both groups when it came to the incidence of diabetes mellitus, overweight and AVA indication. A Cox Regression model with these variables as covariates identified only the HD indication as a positive confounder for the secondary patency rate. Despite this, the saphenous vein as conduit material remained a significant survival advantage. The PTFE group had a significantly higher number of HD AVAs, which may have resulted in an overestimation of the median patency rate.

HD and PN are two different indications for vascular access surgery and both accesses are treated differently. Vascular access for HD is used three to four times a week and allows the patients’ blood to run through an external dialysis device. A vascular access for PN is used up to 7 days a week for intravenous nutrition fluid infusion. Also, the HD AVAs are more frequently evaluated through flow measurements and pressure measurements during haemodialysis sessions. This may result in early detection of AVA failure and makes intervening interventions more effective.

No significant difference was found in secondary patency between HD and PN AVAs with SV. But, despite the use of the anticoagulant medication warfarin in PN patients, the PN AVA with SV tended to occlude more often when compared with the HD AVA.

When conduit materials of PN AVAs were compared, the SV was considered to have a significantly more favourable patency rate compared with PTFE. This may be caused by a high percentage of AVA failure within 3 months for the PTFE conduits. As a result, these findings highlight the use of SV, when possible, if an AVA is needed for PN.

The RCAVF, BCAVF and BBAVF are in this order the first, second and third choice for vascular access when long-term treatment of HD is needed.^11^ As for the fourth option more studies suggest that an autologous interposition is a preferable option when compared with the prosthetic material.^13–17^

This study focuses on the material of the conduit as a factor affecting the patency rate. No doubt other factors are affecting the patency rate, such as comorbidity, age, body mass index, inflammation and medication.

Eventually, all factors affecting the patency should be taken into consideration when vascular access is needed.

## Study limitations

The limitation of the study is the limited size and the heterogeneity of AVA indication in the study population. The other issue is that the choice for conduit material was not randomly. When the SV was insufficient during CDU examination, PTFE materials were used for the AVA. This may have led to a PTFE group with worse superficial venous system quality.

## Conclusion

When a conduit is needed for an AVA and long-term treatment is to be expected, in particular parenteral nutrition, the SV can be preferred over the PTFE as conduit material for a vascular access due to its higher assisted primary- and secondary patency, and lower infection risk.
